# A *Mycobacterium leprae* Hsp65 Mutant as a Candidate for Mitigating Lupus Aggravation in Mice

**DOI:** 10.1371/journal.pone.0024093

**Published:** 2011-09-22

**Authors:** Eliana B. Marengo, Luciana V. de Moraes, Robson L. Melo, Andrea Balan, Beatriz L. Fernandes, Denise V. Tambourgi, Luiz Vicente Rizzo, Osvaldo Augusto Sant'Anna

**Affiliations:** 1 Hospital Israelita Albert Einstein, São Paulo, Brazil; 2 Laboratório de Doenças Genéticas, Instituto Gulbenkian de Ciência, Oeiras, Portugal; 3 Centro de Toxinologia Aplicada-CAT/CEPID, Instituto Butantan, São Paulo, Brazil; 4 Laboratório Nacional de Biociências LNBio, Centro de Pesquisa em Energia e Materiais, Pólo II de Alta Tecnologia, Campinas, Brazil; 5 Laboratório de Imunoquímica, Instituto Butantan, São Paulo, Brazil; Institut Jacques Monod, France

## Abstract

Hsp60 is an abundant and highly conserved family of intracellular molecules. Increased levels of this family of proteins have been observed in the extracellular compartment in chronic inflammation. Administration of *M. leprae* Hsp65 [WT] in [NZBxNZW]F_1_ mice accelerates the Systemic Lupus Erythematosus [SLE] progression whereas the point mutated K^409^A Hsp65 protein delays the disease. Here, the biological effects of *M. leprae* Hsp65 Leader pep and K^409^A pep synthetic peptides, which cover residues 352–371, are presented. Peptides had immunomodulatory effects similar to that observed with their respective proteins on survival and the combined administration of K^409^A+Leader pep or K^409^A pep+WT showed that the mutant forms were able to inhibit the deleterious effect of WT on mortality, indicating the neutralizing potential of the mutant molecules in SLE progression. Molecular modeling showed that replacing Lysine by Alanine affects the electrostatic potential of the 352–371 region. The number of interactions observed for WT is much higher than for Hsp65 K^409^A and mouse Hsp60. The immunomodulatory effects of the point-mutated protein and peptide occurred regardless of the catalytic activity. These findings may be related to the lack of effect on survival when F_1_ mice were inoculated with Hsp60 or K^409^A pep. Our findings indicate the use of point-mutated Hsp65 molecules, such as the K^409^A protein and its corresponding peptide, that may minimize or delay the onset of SLE, representing a new approach to the treatment of autoimmune diseases.

## Introduction

Heat shock proteins [Hsp] are among the most conserved molecules found in prokaryotes and eukaryotes. Intracellular Hsp molecules participate in fundamental cellular processes by acting as chaperones or chaperonins [1], [2]. Under steady state conditions, Hsp molecules represent about 5% of the total intracellular proteins. Under stress conditions this rate may rise significantly [almost 5 times] [3]. The induction of Hsp expression is tightly regulated, as over-expression of this protein may persistent adversely affect the intracellular homeostasis and may lead to cell death [4].

Despite its main function as a chaperone, the participation of the 60 kDa heat shock protein [Hsp60] in chronic-inflammatory processes, including autoimmune diseases, has been widely documented [3], [5], [6]. Heat shock proteins and their derived peptides have been shown to be involved in the pathogenesis of autoimmune diseases such as arthritis, diabetes, and intestinal inflammation as well as encephalomyelitis [reviewed in [6]]. It is believed that the Hsp molecules found in the extracellular compartment play a role in the evolution of autoimmune diseases [7], [8]. In addition, inflammation can significantly increase endogenous Hsp levels, which affect the exposure of cryptic epitopes during antigen presentation and activates an immune response [9].

The fact that in pathophysiological conditions antibodies and T cells may react with self Hsp60 or bacterial Hsp65 suggests that these two proteins are capable of triggering cellular reactions in autoimmune diseases, because the similarity of the bacterial protein and the self antigen [6], [10]. Moreover, increased levels of anti-Hsp60 and anti-Hsp65 antibodies are not restricted to pathological conditions, being also found in healthy individuals [11], [12]. Response to proteins or any peptides should be a naturally occurring subliminal immune phenomenon that participates in the maintenance of neutralization and equilibrium in ordered states aimed to this class of endogenous molecules [13]. Based on the concept of molecular mimicry and on reports suggesting distinct physiological roles for self Hsp60 and bacterial Hsp65 molecules, it has been suggested that variable humoral responses to these proteins may correlate with the occurrence of chronic-degenerative and autoimmune processes [14].

Previously, it has been shown that by adding Hsp to any host an imbalance is observed in both the physiological and the immunological systems. Thus, it was hypothesized that the passive administration of wild type [WT] *M. leprae* Hsp65 interferes with the body endogenous equilibrium by enhancing the entropy of the immunobiological system. Indeed, in a previous study we observed that the severity of Experimental Autoimmune Uveitis [EAU] [15] and the Systemic Lupus Erythematosus [SLE] [16] were increased in mice models that had the wild type *M. leprae* Hsp65 passively administrated. In contrast, the administration of the K^409^A Hsp65, a point-mutated molecule [17], did not affect SLE evolution or survival; the combined administration of WT and K^409^A Hsp65 proteins showed that the K^409^A is able to inhibit but not reverse the effects of WT on F_1_ mice. These data suggest that Hsp65 has a central role in SLE progression, and that the K^409^A may mitigate and delay the development of SLE.

The point mutation of K^409^A *M. leprae* Hsp65 is located at the catalytic site. Thus, it was asked whether disease severity is an effect unrelated to the catalytic function of the WT molecule and whether this same region in either K^409^A or WT Hsp65 molecules play a role on the immunobiological effects associated to mice. To investigate this issue, it was synthesized two peptides that cover residues 352–371 of the *M. leprae* Hsp65 WT peptide [here called Leader pep] and its mutated form [here called K^409^A pep]. It was then observed how each of these peptides impacted the development and progression of SLE in murines. It was found that the immunomodulatory effects caused by each peptide were similar to that caused by their corresponding proteins and that the observed effect occurred regardless of the catalytic activity.

## Results

### Determination of primary structure, synthesis, and characterization of peptides

**Figure 1:**
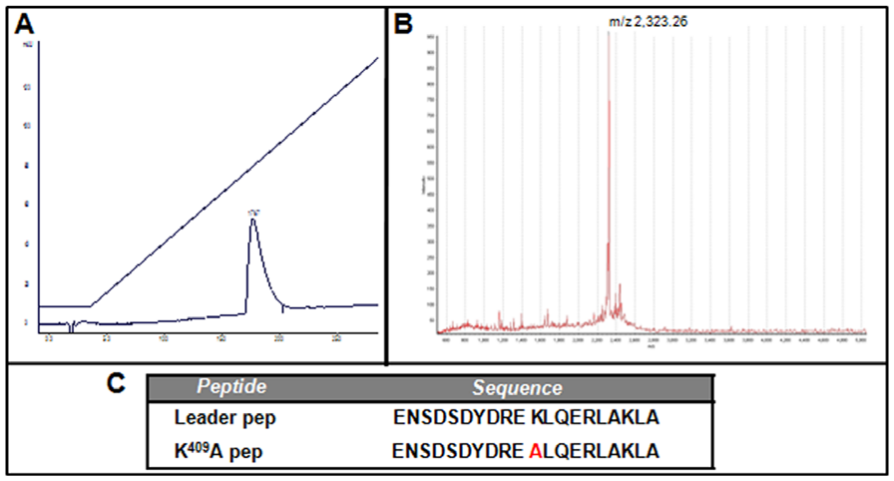
Characterization of K^409^A pep synthetic peptide. A) Purification of K^409^A pep synthetic peptide by reversed-phase chromatography. B) Mass spectrometry analysis of pure K^409^A pep; mz 2,323.26. C) Amino acid sequence of Leader pep and K^409^A pep synthetic peptides.

We have previously shown that administration of *M. leprae* Hsp65 in [NZBxNZW]F_1_ animals accelerated disease progression whereas the point-mutated K^409^A Hsp65 protein had opposite effects in the survival of F_1_ mice [16]. Since mutation was performed at the hypothetical catalytic site [17], we wanted to rule out the possibility that the enzymatic activity of the WT molecule was contributing to disease severity. For this, two synthetic peptides, corresponding to the 352–371 region of the WT and mutant Hsp65 molecules, named here as Leader pep and K^409^A pep respectively, were designed. These molecules comprise only part of the amino acid sequence of the putative catalytic site; in addition, the 352–371 Leader peptide cannot display any catalytic activity because it lacks the minimal structure necessary for an enzyme. Panel A in Figure 1 shows the analytical profile of the K^409^A pep purification after HPLC reverse-phase chromatography. The molecular mass of the K^409^A and Leader peptides corresponded to m/z 2,323.26 and m/z 2,380.54, respectively [Figure 1, panel B]. Panel C [Figure 1] shows the amino acid sequence of the Leader pep and K^409^A pep.

### Immunomodulatory effects of synthetic peptides are similar to those of their respective proteins

**Figure 2:**
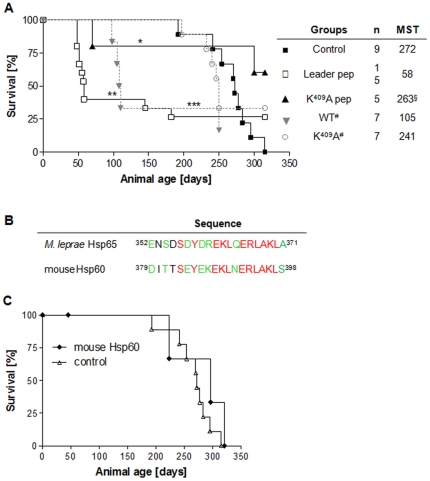
Effect of the Leader pep and K^409^A pep peptides and autologous Hsp60 inoculation on SLE. A) Survival of [NZBxNZW]F_1_ female mice [n = 5 to 15/group] inoculated with synthetic peptides at 45 day-old. §Estimate was limited to the largest censored time [315 day-old]. ^#^Data from [16]. MST = Mean survival time. *p<0.05: K^409^A pep *versus* Leader pep; **p<0.01: Leader pep *versus* control group; ***p<0.001: WT *versus* control group. Results are representative of 2 independent experiments. B) Amino acid alignment of *M. leprae* Hsp65 and mouse Hsp60. Amino acid homology colors: Identical: red; strongly similar: green; different: black. C) Survival of [NZBxNZW]F_1_ 45-day-old female mice [n = 5 to 6] inoculated with mouse Hsp60 [♦]; control group [Δ]. Results are representative of 2 independent experiments.

[NZBxNZW]F_1_ mice of 45 day-old were inoculated with Leader or K^409^A pep and observed for 315 days [which correspond to the maximal survival time of the non-treated mice- control group] or for shorter periods if death occurred earlier than 315 days. Survival of the Leader pep-inoculated animals was significantly affected [*p*<0.01] with approximately 45% [9 out of 15] of mice dying at 53 days of age and showing a mean survival time [MST] of 58 days, as compared to non-treated mice [Figure 2, panel A - MST = 267±35]; 60% [3 out of 5] of the F_1_ mice inoculated with the K^409^A pep survived beyond the age of 315 days. Therefore, estimated survival time of these animals [263 day-old] was limited to the largest censored time, being significantly higher than the Leader pep-inoculated mice [*p*<0.05]. No significant differences were observed between K^409^A pep and control mice, but it is worth mentioning that no animal in the control group reached the age of 315 days. Thus, the inoculation with K^409^A pep may have increased the survival time of these mice. Moreover, peptide treatment generated similar effects on survival of F_1_ mice when compared to their respective WT and mutant Hsp65 proteins. There were no significant differences in response to DNA [titers approximately 7.5log_2_] and to Hsp65 IgG isotypes [titers approximately 4log_2_] in both experimental groups 7 days after the peptides were inoculated.

### Treatment with mouse Hsp60 has no effect on SLE progression

It is hypothesized that the presence of the Hsp60 in the extracellular compartment can propagate the inflammatory response, thereby aggravating autoimmune diseases [6], [18]. In order to evaluate if the mouse Hsp60 has a similar effect to that observed with Hsp65 of *M. leprae* in disease severity, the autologous Hsp60 protein was injected in F_1_ mice. Sequence alignment of the region corresponding to the synthetic peptide *M. leprae* Hsp65 and mouse Hsp60 proteins revealed 55% of sequence identity and the presence of seven amino acids highly conserved [Figure 2, panel B]. No difference in survival times was observed between these groups [Figure 2, panel C].

### Anti-Hsp65 isotype production in H_III_ mice

**Figure 3:**
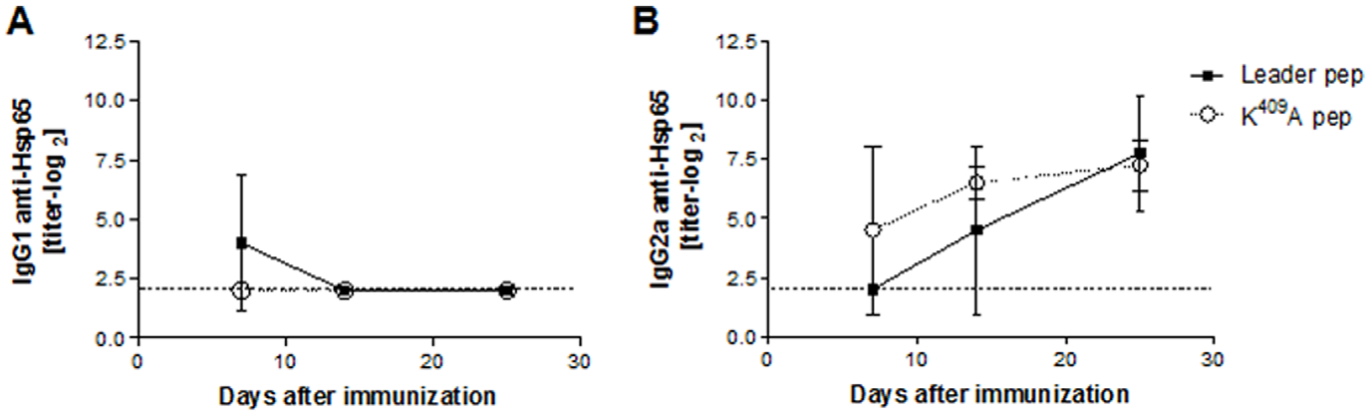
Anti-Hsp65 antibody production in H_III_ mice. Time-course production of anti-Hsp65 IgG1 [A] and IgG2a [B] antibodies in H_III_ mice [n = 3 to 5/group] immunized with Leader pep or K^409^A pep. Antibody titers were evaluated at 7, 14, and 25 days after immunization with synthetic peptides. Results are representative of 2 independent experiments. Dashed lines represent basal levels. Data are expressed as means ± SD.

To evaluate whether Hsp65 synthetic peptides – Leader and Mutant – were immunogenic, H_III_ mice were immunized and the anti-Hsp65 antibody levels at different time-points determined. Basal specific IgG1 antibody levels were observed in all experimental groups [Figure 3, panel A]. IgG2a antibody titers increased in both groups, starting with 4.5log_2_ on day 14 and reaching ∼8log_2_ on day 25 post-immunization [Figure 3, panel B]. These data suggest that Hsp65 peptides are immunogenic even under non-inflammatory conditions, but not pathogenic since H_III_ mice did not develop any apparent illness. Notably, immunization of H_III_ mice with WT *M. leprae* Hsp65, emulsified in IFA, does not induce specific antibody titers [16].

### The inoculation of K^409^A molecule and anti-K^409^A antibodies against Hsp65 mitigates SLE progression

**Figure 4:**
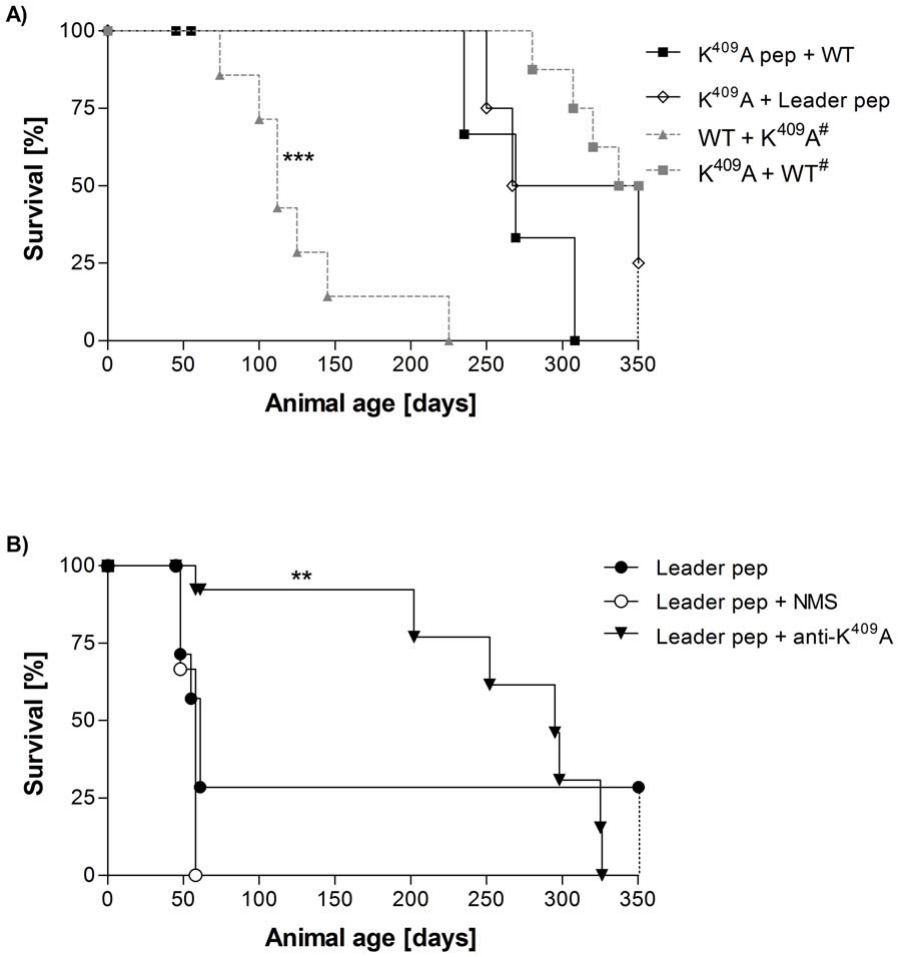
The neutralizing potential of previous inoculation of K^409^A molecule and anti-K^409^A antibodies on SLE suppression. A) Combined effect of K^409^A and WT in survival time. Forty-five-day-old female [NZBxNZW]F_1_ mice [n = 4 to 6/group] were inoculated with K^409^A pep or K^409^A, and after 7 days received WT rHsp65 [K^409^A pep+WT group: ▪] or Leader pep [K^409^A+Leader pep group: ◊]. ^#^Data from [16]. ***p<0.001: K^409^A pep+WT and K^409^A+Leader pep *versus* WT+K^409^A group. B) Survival analysis of [NZBxNZW]F_1_ 45-day-old mice [n = 4 to 7/group] inoculated with Leader pep [•]; Leader pep incubated with H_III_ normal mice sera [○]; or Leader pep incubated with H_III_ K^409^A-immunized mice [▾]. Leader pep group was the reference group for unpaired *t*-test analysis; **p<0.01. Results are representative of 2 independent experiments.

In order to investigate if K^409^A molecules – protein and peptide forms –neutralize the effect of the wild type Hsp65 protein or of the Leader peptide, F_1_ female mice aging forty-five days received K^409^A pep or K^409^A protein and 7 days later were inoculated with the WT molecule or the Leader pep [K^409^A pep+WT or K^409^A+Leader pep groups, respectively]. Survival of the groups that received prior inoculation of mutant peptide or protein forms was significantly enhanced [K^409^A pep+WT group: 271±37 days, and K^409^A+Leader pep group: 289±54 days, *p*<0.001] when compared to groups previously administrated with WT [WT+K^409^A group] or to those that received the Leader pep only. These latter groups showed an MST of 105±10 and 53±4 days, respectively [Figure 4, panels A and B]. The WT+K^409^A pep group was not included in this study because the K^409^A is able to inhibit but not reverse the effect of prior inoculation of rHsp65WT protein on the [NZBxNZW]F_1_ mouse mortality [16].

To further investigate whether anti-K^409^A antibodies inhibit the effect of Leader peptide in SLE, F_1_ animals were inoculated with anti-K^409^A serum produced in H_III_ mice and adsorbed with the Leader pep or normal mouse serum [NMS]. Treatment with anti-K^409^A adsorbed serum resulted in prolonged survival of mice previously inoculated with Leader pep, as compared to controls [Figure 4, panel B], suggesting that anti-K^409^A antibodies neutralize the deleterious effect of the Leader pep.

### Replacement of Lysine for Alanine at position 362 affects the electrostatic potential and interactions in *M. leprae* Hsp65

**Figure 5:**
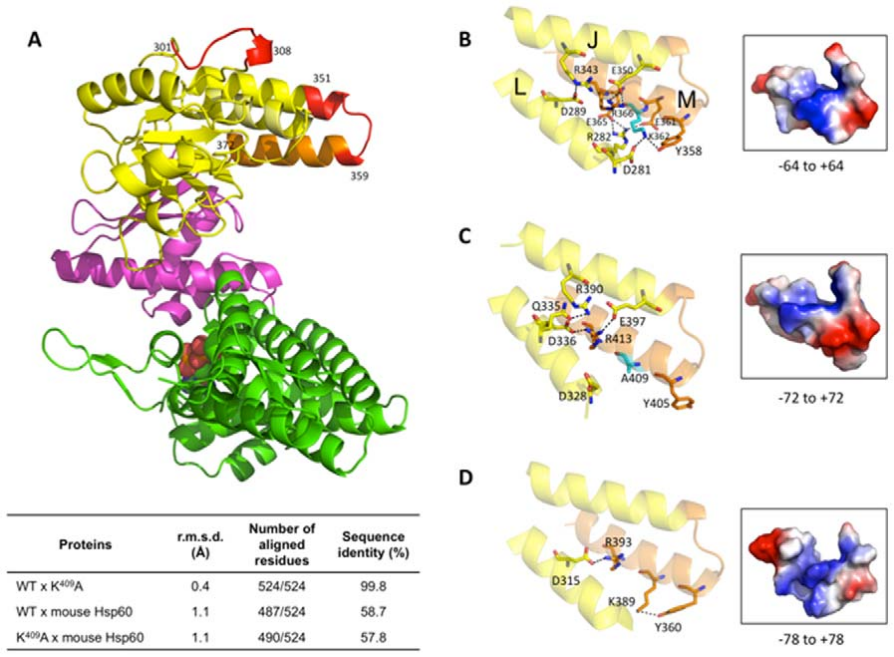
Structural modeling of *M. leprae* WT and K^409^A Hsp65 and mouse Hsp60. A) Superposition of the WT and K^409^A *M. leprae* Hsp65 overall structures showing the three characteristic domains: apical [yellow], intermediate [red], and equatorial [green], with the ADP evidenced in spheres. The peptide region [352 to 371 residues in WT Hsp65] is evidenced in orange and regions with high B-factor described by [21] are shown in red. The inset table shows the results of the structural alignment of the proteins. B, C, and D) Main differences in the polar contacts performed by the peptide region and that affect the electrostatic potential in the WT Hsp65, Hsp65 K^409^A, and mouse Hsp60 proteins, respectively. Red color shows negative and blue positive charges.

In order to better understand the effects of Hsp65 WT and K^409^A protein- treatment in F_1_ mice, structural models of both molecules were built. WT *M. leprae* Hsp65 and mutant were built based on the structural coordinates from *M. tuberculosis* Hsp65 [PDB code 1SJP_A] [19], which shows 97.7% of identity with the *M. leprae* Hsp65. The stereochemical quality of the models was evaluated with Procheck and the Ramachandran plot revealed 94.5% and 96% of residues in most favored regions for WT Hsp65 and mutant, respectively. No residues were found in generously allowed or disallowed regions in both models [data not shown]. For comparison, the mouse Hsp60 was also modeled using the structural coordinates of the chaperonin GroEL from *E. coli* [PDB code 1SX3] [20], which has 50% of amino acid sequence identity. Ramachandran plot for this model showed 94.8% of residues in most favored regions and none in generously or disallowed regions [data not shown]. All the models presented the three characteristic apical, intermediate, and equatorial domains found in *E. coli* Hsp60 [Figure 5, panel A, colored in yellow, magenta, and green, respectively] [21]. The peptide region is located in the apical domain [colored in orange and red], forming a “handle” that is very exposed and which does not interact with the ATP ligand-binding site in the equatorial domain [shown in red spheres]. In addition, based on the rigid model data, the substitution *of Lys 362 by Ala* does not modify the alpha helix arrangement in the 352–371 region. The structural superposition of the three proteins revealed no significant differences, measured by the root mean square deviation [r.m.s.d.] for the á-carbon. Values varied from 0.4 Å [WT *versus* K^409^A] to 1.1 Å [WT/K^409^A *versus* mouse Hsp60] [Figure 5, inset table]. On the other hand, there are significant changes in the polar contacts and electrostatic potential of the peptide region, when compared among the three proteins. The position of the fragment covering residues 352 to 371 in the WT Hsp65 protein allows more inter alpha-helices *M* and *J* interactions [Figure 5, panel B and Table 1], than the same fragment in the Hsp65 K^409^A and mouse Hsp60 [Figure 5, panels C and D, respectively, and Table 1]. According to this model, in the WT Hsp65, residues Y358, E361, K362, E365, and R366, [all of them located in the M helix in the 352–371 peptide region] compose eight contacts connecting helices *J* and *L*, including two contacts performed by the Lysine 362 [K362/NZ – D281/OD2, 2.8 Å and K362/NZ – Y358/OH, 2.9 Å; Table 1]. When Lysine is replaced by Alanine [K^409^A mutant], only the R413, equivalent to the R366 in the WT Hsp65, has contacts with D336 of the *L* helix and E397 [*J* helix].

**Table 1:** Polar contacts identified in the three models built for WT Hsp65, Hsp65 K^409^A, and mouse Hsp60.

WT Hsp65			Hsp65 K^409^A			mouse Hsp60		
residue 1	residue 2	distance (A)	residue 1	residue 2	distance (A)	residue 1	residue 2	distance (A)
R343/NZ	D289/OD1	2.7	E397/OE1	R413/NH1	2.7	R393/NH1	D315/OD2	2.8
E350/OE2	R366/NH2	2.8	D336/OD2	R413/NH2	2.7	**K389/NZ**	**Y385/OH**	**3.1**
E350/OE2	R366/NH1	2.7	R390/NH1	Q335/OE1	2.8			
R282/NH1	E365/OE2	2.8						
R282/NH2	E365/OE2	2.8						
R282/NH1	E361/OE2	3.0						
**K362/NZ**	**D281/OD2**	**2.8**						
**K362/NZ**	**Y358/OH**	**2.9**						

The region showed refers to the residues 352 to 371, according to the number of residues of the WT Hsp65 protein. Contacts in bold are those performed by K362 in WT Hsp65 and K389 in mouse Hsp60.

A slight increasing of the electrostatic potential is observed when the 352–371 fragment of both WT and mutant molecules are compared [Figure 5, panels B and C]. This may have some effect on the folding and structure of the *M. leprae* Hsp65. Curiously, although the mouse Hsp60 conserves the Lysine at the same position found in WT Hsp65, the number of interactions of the peptide region in this protein follows the profile of K^409^A mutant Hsp and is similarly decreased [Figure 5, panel D and Table 1]. The amino acid hydropathicity and polarity of the peptide region in the three proteins was compared using Protscale server [22], according to [23] and [24], respectively. WT Hsp65 and K^409^A showed similar profiles for polarity but an increased hydropathicity in Hsp65 K^409^A, which is comparable to that calculated for mouse Hsp60, was observed. In addition, mouse Hsp60, which shares only 55% of sequence identity with WT Hsp65, presented high polarity in the first eight residues of the 379–398 fragment [data not shown].

### Distinct potential binding of the 352–371 amino acid regions of WT and K^409^A proteins to MHC class I and II

**Figure 6:**
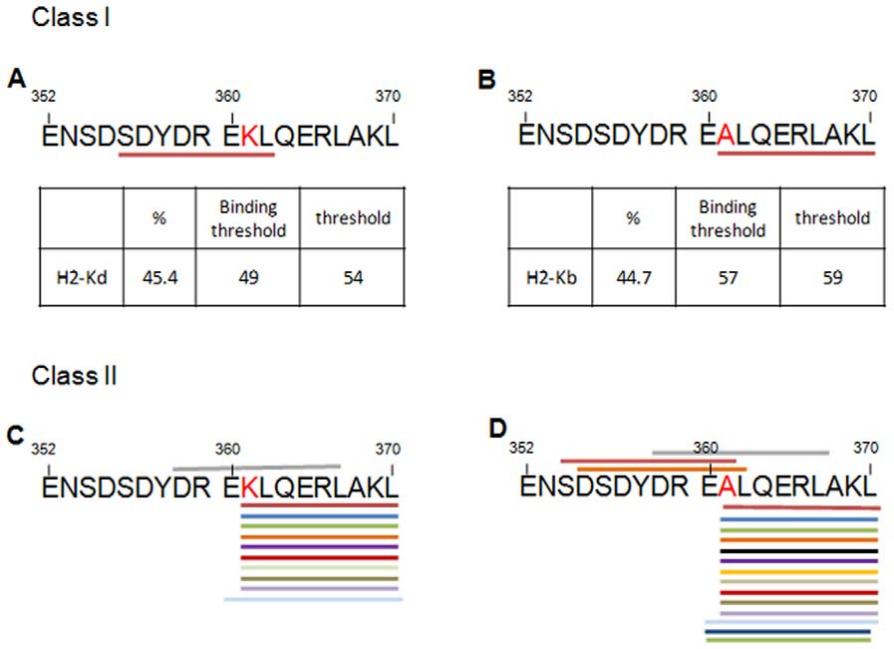
Analysis of the prediction of peptide-epitope binding to MHC class I and II molecules for WT and K^409^A *M. leprae* Hsp65, focusing in 352–370 amino acid region. Theoretical amino acid sequence identification of peptide-epitopes of the WT (A) and K^409^A (B) Hsp65 and the homology percentage of consensus peptide binders to MHC H2 alleles. Primary structure identification of peptide-epitopes of the WT (C) and K^409^A (D) binding to mice and human MHC class II molecules.

Theoretical analysis evidenced that for the MHC class I only 1 out of 10 alleles of the H-2 molecule available for the RankPep recognizes the 352–371 region of WT and K^409^A. The recognition ratio with the consensus sequences of each molecule of MHC class I was about 45% for both proteins [Figure 6 – panel A and B]. However, distinct class I molecules were predicted to bind to the 352–371 amino acid region of the Hsp65 wild-type and mutant molecules: the H-2^kd^ allele recognized the SDYDREKL peptide-epitope of WT [Figure 6 – panel A] and the H-2^kb^ allele recognized the ALQERLAKL of the mutant protein [Figure 6 – panel B]. For the MHC class II, 12 and 17 potential MHC peptide-binders to WT and K^409^A molecules were respectively identified. There are 12 H-2 alleles and 49 HLA sub-alleles available at RankPep. While, the wild type and mutant proteins share these same alleles for class II, five additional HLA alleles that recognized the 352–371 region of K^409^A exist [Figure 6 – panel D].

## Discussion

Motivated by the opposite immunomodulatory effects of WT and K^409^A Hsp65 on SLE, we analyzed the synthetic peptides corresponding to the region of the K^409^A mutation in [NZBxNZW]F_1_ mice, a model of spontaneous Systemic Lupus Erythematosus. Following our previously concepted system based on the addition of Hsp to imbalance the endogenous equilibrium of the immunobiological system [16], the Leader and K^409^A peptides were passively administered to these F_1_ mice. Here the primary sequence of synthetic peptides derived from *M. leprae* Hsp65 and their effects in this model are presented. The present data showed that, in general, the action of these synthetic peptides resembled that of their respective proteins, indicating that the residues 352–371 of both *M. leprae* Hsp65 and of the mutated form K^409^A are the ones responsible for the effects on mice survival. This rules out the hypothesis that the immunomodulatory effect previously observed [16] was associated with a catalytic activity of the *M. leprae* Hsp65. Furthermore, the effect of each peptide was comparable to that observed in its corresponding proteins. Not only that but this effect was also amplified. In other words, there is a reduction in the survival time of mice injected with the Leader pep, and a tendency for increased survival time with K^409^A pep inoculation, as compared to the survival times observed with the two corresponding proteins. Although there were no significant differences in survival times between K^409^A pep and the control group, a lower standard deviation in K^409^A pep-inoculated animals was observed, suggesting that this peptide could increase the survival of animals that would otherwise have an early death, possibly being the ones with more severe disease. Similar observations have been made previously in lupus animals inoculated with the K^409^A Hsp65 protein [16].

Because the administration of K^409^A protein as well as K^409^A pep had no effect on mortality of [NZBxNZW]F_1_ and the K^409^A protein presents higher immunogenicity than the WT Hsp65 [16], the effects of the co-administration of K^409^A+Leader pep or K^409^A pep+WT on the survival of F_1_ animals were evaluated. The survival times of K^409^A+Leader pep and K^409^A pep+WT animals were significantly higher when compared to Leader pep or WT-inoculated animals. These results indicate that the K^409^A protein and its corresponding synthetic peptide are able to inhibit the deleterious effect of the Leader pep or WT on the mortality of the [NZBxNZW]F_1_ mouse. Indeed, the transfer of the anti-K^409^A serum produced in H_III_ mice and adsorbed with the Leader pep reinforces the mitigating effects of K^409^A forms in SLE. Altogether, these data indicate the neutralizing potential of prior inoculation of K^409^A molecule, both protein and its peptide, and anti-K^409^A antibodies against Hsp65, and possibly against autologous Hsp60. Further protein–protein interaction studies will expand our knowledge on the potential therapeutic application of K^409^A forms on lupus disease, and possibly on other chronic degenerative autoimmune processes.

Despite the various pro-inflammatory properties described for Hsp60 family members [25], [26], [27], [28], other reports support an immune regulatory role for self Hsp60 [29], [30], [31], [32], [33]. Here, the administration of autologous Hsp60 protein did not change survival of F_1_ animals. Thus, it is possible that in this model the recombinant Hsp60 does not activate immune cells; otherwise the molecule would be recognized as a self-antigen. However, it is possible that administration of higher doses of autologous Hsp60 [>2.5 µg] interferes with lupus progression.

The sequence alignment analysis of 352–371 amino acids of *M. leprae* Hsp65 and the corresponding 379–398 of mouse Hsp60 shows 55% of identity and an additional of 30% of strongly conserved amino acids. The literature shows that the amino acid differences between mouse Hsp60 and bacterial Hsp65 include the less conserved regions of these molecules, which are recognized by vertebrate T cells [34]. Taken together, these observations reinforce the idea of a higher immunogenic property of *M. leprae* Hsp65 as compared to that of autologous Hsp60 in disease aggravation. They also suggest a lack of cross-reactivity of conserved epitopes corresponding to the synthetic peptide between mycobacterial Hsp65 and mouse Hsp60. This finding supports the view that the structural similarity of the epitopes, based on the anchor residues, is more important than the homology of the primary sequence.

Several crystal structures of GroEL – *E. coli* Hsp60 chaperonin 1 [cpn60-1] and from other organisms are available and two of them show GroEL in its 14-meric structure [21], [35]. For the mycobacterial Hsp65, the crystal structure of Hsp65 *M. tuberculosis* reveals its dimeric state and suggests that the concentration of Hsp65 found in the extracellular milieu is extremely low for oligomer formation [19], [36]. Also, the differences in the primary sequence of the two chaperonins [cpn60] homologs of *M. leprae* – cpn60-1 [Hsp60] and cpn60-2 [Hsp65] – may contribute to the divergent structural and functional properties observed in the Hsp60 family members.

Here, the tertiary structure of *M. leprae* Hsp65 and its mutant K^409^A were based on templates of crystal monomer structure of the *M. tuberculosis* Hsp65 [PDB code 1SJP] [19] and of the chaperonin complex from *Thermus thermophilus* [PDB code 1WE3] [37]. No significant changes in the tertiary structure of both proteins, including the predicted alpha-helix where the mutated Lysine K^409^A is located, were observed. However, although the WT and mutant Hsp65 are expressed as rigid structures based on a model that fixes the positions of amino acids, replacing Lysine 362 by Alanine affects the electrostatic potential of the region and the potential interactions performed by Lysine 362 and the residues located around it. Importantly, the mutation at the 352–371 region probably does not affect the molecular stability, regardless whether it is in its heptamer or monomer structures, because this region is distantly located from the ATP binding site. In addition, the number of interactions observed at this site of the WT Hsp65 is highly decreased when compared to mouse Hsp60 and Hsp65 K^409^A; both these proteins showed a similar hydrophobic profile for the equivalent 352–371 region of Hsp65. These features could be related to the lack of effect and enhancement of survival of F_1_ mice when they were inoculated with autologous Hsp60 or K^409^A pep, respectively. As mentioned, because the models are shown as rigid structures, it is possible that the differences in the peptide region affect the positions and interactions between *M* and *J* helices. Interestingly, one of the regions evidenced by [21] that shows the high B-factor of the predicted protein structures, and is located exactly in the most exposed area of the peptide region [Figure 5, panel A], is related to a loss of interactions between the helices that are associated to the higher mobility of the apical region. This could lead to drastic effects on the 352–371 peptide region and protein of the K^409^A Hsp65 structure and could change the interaction with other molecules.

The structural model shows that the 352–371 region of the Hsp65 is exposed, and it is possible that it can be recognized by the immune system. Our current data from immunization of HIII mice with the Leader or K^409^A peptides show that, in general, the synthetic peptides seem to be more immunogenic when compared to their corresponding proteins [16], especially for the wild type protein form. This may be related to the short extension of the peptides, which may be more easily processed and presented by the immune system than their respective proteins. A group of small peptides and a 20.4 kDa fragment of the C-terminal portion from Hsp65, commonly found on the cell wall of *M. leprae*, are in the cytosol of the bacillus [38]. *In vitro* studies also show a potential autolysis of *M. leprae* Hsp65 by releasing portions of its N- and C-terminal regions, suggesting that the antigen presentation occurs independently of the proteasoma [39]. The process of chaperone/chaperonin autolysis has also been described for other Hsp families and it is believed that self-degradation is a modulation process [39]. Therefore, the inflammation observed in chronic inflammatory processes, including autoimmune diseases, may change the conformation, the antigen processing, or self-degradation of the Hsp65. This hypothesis is supported by a recent paper from [40]. Inflammation may also expose or more efficiently release new determinants/epitopes, or certain epitopes such as the cryptic ones [18], and possibly the region that comprises the 352–371 peptide.

Discrimination between the pathological and regulatory Hsp60 actions in autoimmunity is still unclear, even whether the regions of the Hsp60 may determine their opposing immunologial function. However, some reports suggest that different regions of the Hsp60 molecules induces distinct immune response; proliferative response induced by N-terminal and intermediate Hsp60 peptides, which also induced IFN-γ production; also, the IL-4 production was induced by the intermediate and C-terminal regions [30]. It was reported that a C-terminal region of Hsp60 [354–366 amino acid] was involved in LPS binding and innate immune activation [41]. On the other hand, the p277 peptide from 437–460 amino acids of the human Hsp60 molecule presented immunoregulatory effect in non-obese diabetic NOD mice, by inducing IL-4 and IL-10 production [42], [43].

Although there were no significant differences in the production of anti-DNA and anti-Hsp65 IgG isotypes it is possible that these antibodies differ in their binding affinity, leading to distinctive potential pathogenic involvements. In the present model, a preliminary analysis indicates that in mice receiving WT or K^409^A proteins, the avidity was ten times higher than their respective peptides and controls at 80-day-old mice [data not shown].

The exact pathophysiology mechanism behind the phenomenon evoked by the K^409^A has not been identified. Preliminary results showed that the K^409^A-inoculated lupus mice presented lower number of lymphocytes in spleen when compared to WT mice group and controls and that this effect is not mediated by direct induction of apoptosis evaluated *in vitro* at 2 and 24 hours. Furthermore, total spleen cells treated with K^409^A protein or its peptide indicate that these mutant forms did not affect *in vitro* cell proliferation assay. Thus, it can be suggested that modulation rather than activation is what occurs in the immune system. In addition, no differences in frequency of *ex vivo* CD4^+^CD25^+^ and CD4^+^CD122^+^ populations were observed. Neither difference was observed in IL-2 cytokine production in splenic cells in WT and mutant groups when compared to controls. Also, no differences in splenic IL-10 levels were observed [unpublished data]. It is possible that, at least in part, the immunomodulatory effect of the K^409^A protein/peptide is explained by a balanced production of IgG1 and IgG2a anti-DNA along the life and high levels of humoral IFN-γ in this experimental group when compared to WT and control groups [16]. In a systemic and multifactorial chronic disease such as SLE, it must be considered that there is not a unique explanation and assertion, since competing and overlapping mechanisms occur in different compartments and at the same time.

The discordant results obtained *in vivo* with WT and K^409^A forms raise some questions, considering that the protein and the peptide are point-mutated molecules. The analyses of RANKPEP prediction of peptide-epitope of WT and K^409^A Hsp65 were relatively distinct in their potential to bind to MHC molecules. Therefore, it can be hypothesized that the MHC Hsp65-binding potential is one of the features responsible, at least in part, for the effects that Hsp65 causes in the survival of F_1_ mice. Because the concomitant administration of the K^409^A pep and Leader pep in lupus mice had a predominant effect of the mutant peptide, we assume that they compete for the same target on molecular and cellular levels, such as antigen presenting cells and MHC molecules. This hypothesis deserves further investigation.

As shown in our studies, a single base change in the DNA can have remarkable effects, such as those artificial changed molecules observed for the altered peptide ligands [APL]. There are reports in the literature of natural variants or mutations of *M. leprae* Hsp65 [44]. Additionally, amplification of some regions of the *M. leprae* Hsp65 may be used for a differential diagnosis of leprosy [45]. Nevertheless, the molecular and cellular mechanisms caused by these variations in the *M. leprae* Hsp65 in infected individuals remain unknown.

Our findings suggest that the use of point-mutated molecules, such as the K^409^A protein and its corresponding peptide, may minimize or delay the onset of SLE, representing a new possibility of treatment for this and other autoimmune diseases.

Evolutionarily, adaptations towards the functional aspects of the proteins might have been initially predominant and, along eras, associated to the progressive organism complexity, as structural diversities were fixed. Distinct functions, binding motifs, and molecular interactions are reflected in conserved families, such as those of the immunoglobulins or of the heat shock proteins. Thus, the molecular evolution would be defined by the capacity of the combination potential between molecules, their affinity, and molecular conformation in the *lato* sense. Relative to energy dissipation, it can be assumed that during the evolutionary process, the best intermolecular adjustment determines species survival. The molecular relationships and the cellular interactions can be understood as actions defined for balance and sequential reactions. The dynamics of the complex network that delineates the immune system, and also the complex toxicity processes, can be included in the contexts of imbalance of dissipative structures and ordered disorganization, which are guided by causal and extemporal sequences. It must take into account that the perception of the significance of the concept of self/non-self is limited by possibilities of detection or not, of binding to or neo-exposition of pre-existing molecules. To the qualitative network emergence must be adjoined the extremely variable and complex quantitative and pleiotropic expression of responsiveness. For most of the physiological processes that are cumulative and irreversible [mainly those related to the immune system] it is possible that even though irregularities, a variety of subliminal and undetected pathological processes could be initiating. Based on these and our previous data, Hsp could be understood as toxins. Immunities or toxins, behaving as a broken mirror in which similarities or contrasts acquire variable structures and expressions along the individual life, give surety to species survival.

## Materials and Methods

### Animals

Mice were caged and handled under ethical conditions, according to international rules of animal care specified in the International Animal Welfare Recommendations [46]. New Zealand Black [NZB] female and New Zealand White [NZW] male mice were obtained from the animal facilities of the University of São Paulo, Brazil and from the Gonçalo Moniz Research Center, Salvador, Bahia, Brazil. The genetically selected high responder mice [H_III_ line] were obtained from the Immunogenetics Laboratory of Instituto Butantan, São Paulo, Brazil. Parental lines were mated in our laboratory to produce [NZBxNZW]F_1_ hybrids. After weaning, F_1_ mice were housed in groups of four to six animals in plastic cages filled with hardwood bedding, and were provided with water and rodent feed *ad libitum*. The animals were kept in a room with controlled lighting [12-h light/dark cycle], pressure, humidity, and temperature [24°C]. All the procedures were approved by Institutional Animal Care Committee at Butantan Institute [CEUAIB # 196/05].

### Peptide synthesis

Leader pep [ENSDSDYDREKLQERLAKLA] of *M. leprae* Hsp65 and K^409^A pep [ENSDSDYDREALQERLAKLA] of the mutated form K^409^A, both covering residues 352–371, were synthesized using the Fmoc [*N*-(9-fluorenyl)methoxycarbonyl) procedure [47] in a Shimadzu PSSM-8 peptide synthesizer [Shimadzu, Tokyo, Japan]. The Fmoc-amino acids were purchased from Novabiochem [Nottingham, UK]. The synthetic peptides were purified by preparative reversed-phase chromatography [reversed-phase HPLC], and the purity and identity of the peptides were confirmed by matrix-assisted laser desorption ionization time-of-flight [MALDI-TOF] mass spectrometry on Ettan MALDI-TOF/Pro instrument [Amersham Biosciences, Buckinghamshire, UK] and by analytical reversed-phase high performance liquid chromatography [HPLC] [Shimadzu Inc., Tokyo, Japan].

### Expression of the recombinant *M. leprae* Hsp65 in Escherichia coli

Expression and purification of the recombinants *M. leprae* WT and K^409^A Hsp65 was done as described in [16].

### Animal treatment

Forty-five-day-old female [NZBxNZW]F_1_ mice were intraperitoneally [i.p.] inoculated with a single dose of 2.5 µg of Leader pep or K^409^A pep in 0.2 ml of phosphate buffer saline pH7.4 [PBS]. An additional group of animals was inoculated with 2.5 µg of mouse Hsp60 [Stressgen, ESP-741D] in 0.2 ml of PBS. The number of animals varied between 5 and 15 per group and control mice received 0.2 ml of PBS. Peptide-inoculated and control mice were periodically bled and the individual serum samples stored at −20°C until titration by ELISA of the anti–DNA and anti–Hsp65 antibodies. Animals were evaluated until 315 days of age or until their death. Mice were periodically examined for clinical signs including development of ascites, lethargy, anorexia, and death.

### Anti-Hsp65 antibody production in High responder mice

Three- to four-month-old H_III_ mice were immunized subcutaneously with 10 *μ*g of WT rHsp65, K^409^A, Leader pep, or K^409^A pep emulsified in incomplete Freund's adjuvant [IFA] [v/v] in a final volume of 200 *μ*L. These high responder mice were genetically selected according to high responsiveness and expressed no epitope nor isotype restrictions [48], [49]. For the recombinant Hsp65 proteins, after approximately 15 days of immunization [primary response], animals received a subcutaneous booster of 10 *μ*g of the respective recombinant proteins emulsified in IFA [v/v] in a final volume of 200 *μ*L. Mice were periodically bled by retro-orbital venous plexus and the samples collected were kept at −20°C for further analysis of individual primary and secondary responses.

### *In vitro* and *in vivo* sera assays

The *in vitro and in vivo* neutralizing sera assays were carried out as follows: 2.5 µg of Leader pep were incubated for 30 minutes at 37°C with serum anti-K^409^A produced in H_III_ mice [as described above] or H_III_ normal mice serum [NMS] at 1∶4 dilution. Mixtures were centrifuged at 14000 rpm for 10 minutes at 4°C, and the supernatants were collected and inoculated in [NZBxNZW]F_1_ female mice [n = 4–7/group] with 45 days of age by the intraperitoneal route. As control, F_1_ mice were inoculated with 2.5 µg of Leader pep. Clinical signs, including ascites development, lethargy, and anorexia, as well as the mean survival time were evaluated.

### Combined administration of recombinant Hsp65 and synthetic peptides

Forty-five-day-old female [NZBxNZW]F_1_ mice were inoculated i.p. with a single dose of 2.5 µg of K^409^A pep or K^409^A protein in 0.2 ml of PBS. Seven days later, animals received 2.5 µg of WT rHsp65 or Leader pep, i.p. Mice were periodically bled; individual serum sample titration and clinical signs were evaluated as described above.

### Titration of anti-DNA and anti-Hsp65 antibodies

Specific IgG1 and IgG2a isotypes were detected with indirect ELISA as described in [16].

### Molecular modelling of the tridimensional structures of *M. leprae* Hsp65 and mouse Hsp60

The structural models of *M. leprae* WT and K^409^A mutant Hsp65 and mouse Hsp60 were generated using the Modeller 9v2 program [50], driven by satisfaction of spatial restraints, using the protein sequences P09239 and P63038, respectively. Search for best models were performed using FUGUE [51] and PSI-Blast against Protein Data Bank as a search set. The mouse Hsp60 tridimensional model was generated based on the atomic coordinates of the *Escherichia coli* chaperonin GroEL [PDB code 1SX3] [20], which shares 50% of sequence identity. For building of *M. leprae* Hsp65 models, two proteins were used. The atomic coordinates from the chaperonin complex from *Thermus thermophilus*, chain A [37] were used for modelling of N and C termini [PDB code 1WE3, residues 3 to 70 and 510 to 529, respectively] and the structural coordinates of the Hsp65 from *M. tuberculosis* [PDB code 1SJP, residues 60 to 514] [19]. The best models were chosen according to the Modeller objective function and stereo chemical analyses using Procheck [52]. Angle distortions and rotamers were corrected using COOT [53]. Secondary structure matching [SSM] superposition of the models was obtained according to [54]. All the figures and the electrostatic potential calculation were obtained with “The PyMOL Molecular Graphics System” [55].

### Alignment of Hsp60 amino acid sequences

The primary structure analysis of the mouse Hsp60 and *Mycobacterium leprae* Hsp65 was performed using the Clustal W – multiple alignment website [http://npsa-pbil.ibcp.fr], supplied with sequences deposited in the SWISS-PROT/TrEMBL database [http://expasy.org/sprot], access number: *M. leprae* Hsp65: P09239; mouse Hsp60: P63038.

### Major Histocompatibility Complex [MHC] class I and II epitope binding prediction

The potential binding of the 352–371 amino acid regions of WT and K^409^A proteins to MHC class I and II was analyzed using the RankPep tool. Theoretical recognition ratio in the consensus sequences of each molecule of MHC class I and II and the number of the molecules that recognize the peptides were calculated for both proteins.

### Statistical analysis

Antibody production data are expressed as the mean [X] ± standard deviation [SD] and compared by unpaired *t*-test. Survival time was analyzed by Kaplan-Meier curves and log-rank test. Statistical significance was set at *p*<0.05.
